# Structural and functional characterization of IdeC, a novel IgG-specific protease of *Streptococcus canis*

**DOI:** 10.1128/iai.00248-25

**Published:** 2025-07-31

**Authors:** Saoirse Walsh, Antje-Maria Lapschies, Vega Miguel-Ruano, María T. Batuecas, Iván Acebrón-Ávalos, Thomas P. Kohler, Sven Hammerschmidt, Inga Eichhorn, Juan A. Hermoso, Marcus Fulde

**Affiliations:** 1 Institute of Microbiology and Epizootics, School of Veterinary Medicine, Freie Universität Berlin, Berlin, Germany; 2 Department of Crystallography and Structural Biology, Institute of Physical-Chemistry "Blas Cabrera", CSIC 69568, Madrid, Spain; 3 Centre for Functional Genomics of Microbes, Universität Greifswald 26552, Greifswald, Germany; 4 Robert Koch Institute, Genome Competence Centre (MF1) 9222, Berlin, Germany; Washington State University, Pullman, Washington, USA

**Keywords:** host pathogen interaction, *Streptococcus canis*, Immunoglobulin G, IgG protease, host specificity

## Abstract

*Streptococcus canis* is an important opportunistic pathogen of cats, dogs, and cows, which can cause a range of infections, ranging from skin and soft tissue infections to septicemia and endocarditis. As a zoonotic agent, *S. canis* has also recently been implicated in serious human infections, following trauma or immunosuppression. In this work, we describe a novel protease of *S. canis*, termed IdeC (Immunoglobulin G degrading enzyme of *S. canis*), which may be involved in bacterial immune evasion. The cleaving ability of IdeC against IgG from various species was assessed; this revealed that IdeC successfully cleaved canine, feline, and human IgG. We also confirmed that IdeC is a cysteine protease, similar to IdeS of *Streptococcus pyogenes*. Investigation of the cleavage site in IgG sequences showed that it is highly conserved across IgGs from all species tested. From this analysis, it was determined that IdeC cleavage occurs between the CH2 and hinge regions of IgG. Interestingly, feline IgG was consistently cleaved with the highest efficiency, with human and canine IgG displaying less efficient cleavage. High-resolution crystal structures of two IdeC constructs provided insights into the catalytic machinery and substrate recognition. Modeling of the full-length IdeC:IgG complexes for human, canine, and feline cases explains the mechanism of action of the protease and reveals the molecular basis for the observed cleavage preference for feline IgG. Understanding and managing *S. canis* as a pathogen is important in both veterinary and human medicine, as this bacterium underscores the need for awareness of zoonotic transmission.

## INTRODUCTION

*Streptococcus canis* is a gram-positive, β-hemolytic coccus that can belong to Lancefield groups G or C (1, 2). It is largely a commensal bacterium of dogs and cats, which can act as an opportunistic pathogen mostly causing infections of the skin and soft tissue, although also capable of causing more severe diseases like endocarditis (3, 4). Although initially thought to affect only dogs and cows (1), *S. canis* has now been isolated from a wide range of hosts, with dogs and cats being most prevalent (5). Human infections have also been recorded (6). Reported infections in humans have been connected to severe diseases such as septicemia (7–9).

Understanding the interplay between pathogens and the host immune system can provide insight useful to infection control and disease prevention. Immunoglobulins are a vital component of the adaptive immune system, responsible for recognizing a wide variety of antigens and, subsequently, induction of immunological effector mechanisms (10). Immunoglobulins are composed of the variable/antigen-binding Fab region and the constant/effector Fc region, joined by a flexible hinge region (11). This hinge region is the cleavage site for proteins such as papain and pepsin (12). Upon antigen recognition by the Fab region, the Fc region activates the classical complement pathway via complement component C1q (11). Humans have five main isotypes of immunoglobulins: IgG, IgD, IgA, IgE, and IgM. IgG is the most abundant in humans (10) and dogs (13). In addition to complement activation, IgG can also bind to bacteria, marking them for opsonization by phagocytic cells (11).

Several streptococcal proteases have been described, including IdeS/MAC and SpeB of *Streptococcus pyogenes* (14), IdeP of *Streptococcus phocae* subsp. *phocae* (15), IdgE of *Streptococcus suis* (16), IdeE of *Streptococcus equi* subsp. *equi (17*), and IdeZ, IdeE2, and IdeZ2 of *Streptococcus equi* subsp. z*ooepidemicus* (17, 18). All of these have been demonstrated to cleave immunoglobulins, thereby aiding in immune evasion. IdeS, IdeP, and IgdE have been further characterized as cysteine proteases (14–16), characterized by a cysteine residue at their active site that acts as a nucleophile during catalysis (19). The cysteine protease catalytic triad of Cys/His/Asn or Asp is vital to the catalytic function of the protein (20). Bacterial cysteine proteases are involved in a broad range of processes, including infection (20).

This study describes a novel cysteine protease of *S. canis* designated IdeC (Immunoglobulin G degrading enzyme of *S. canis*) and investigates the immunoglobulin cleaving abilities of this protein.

## RESULTS

### Identification of a novel streptococcal protease

**Fig 1:**
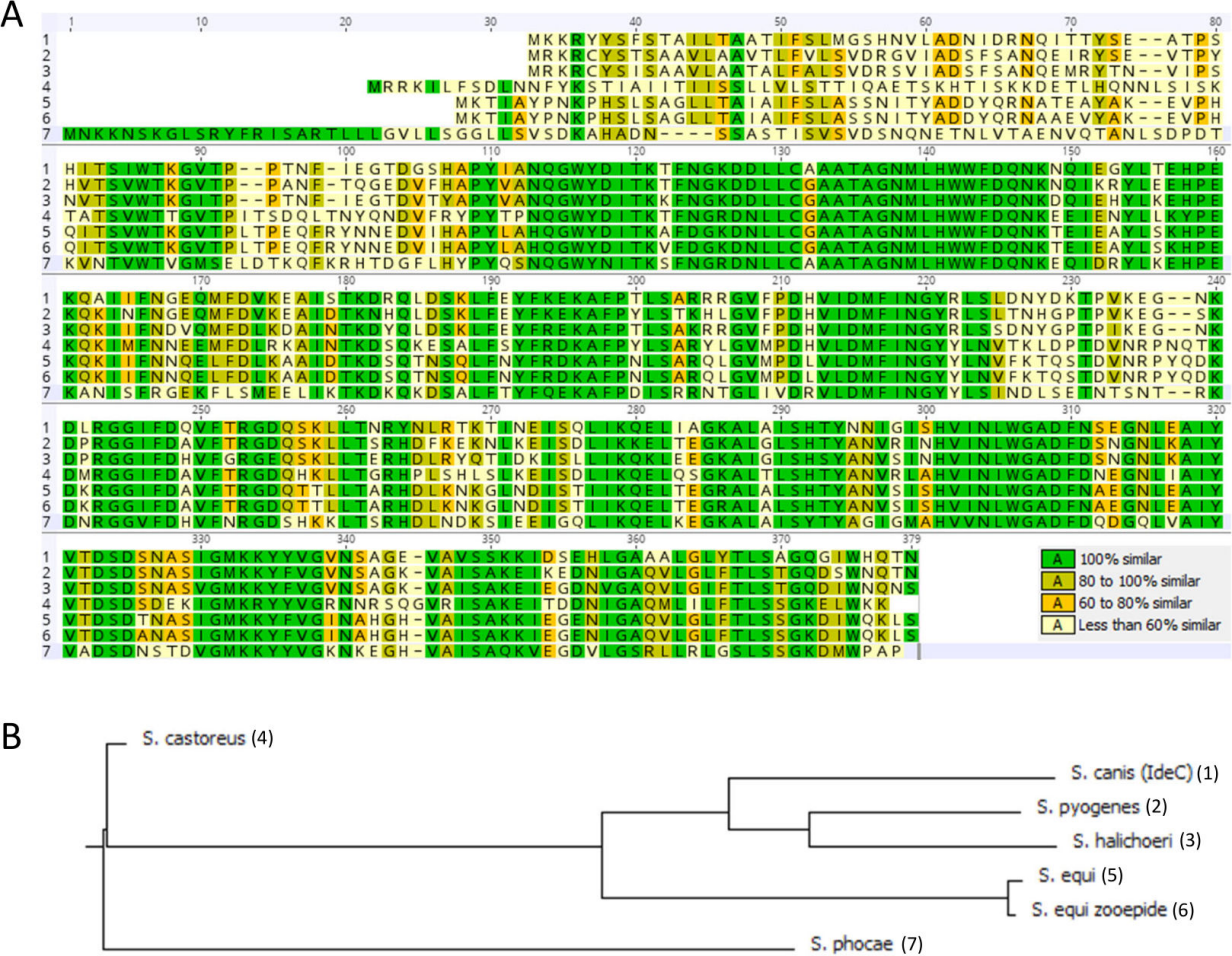
Sequence alignment of cysteine proteases of streptococcal species. (**A**) Sequence comparison of streptococcal cysteine proteases, created in Geneious 11.1.5. Included proteins are (including NCBI accession number) (1); IdeS/Mac family cysteine endopeptidase, WP_003044545 (*S. canis*) (2), Immunoglobulin G-degrading enzyme IdeS, WP_032462089 (*S. pyogenes*) (3), IdeS/Mac family cysteine endopeptidase, WP_159797190.1 (*S. halichoeri*) (4), IdeS/Mac family cysteine endopeptidase, WP_051188344 (*S. castoreus*) (5), IdeS/Mac family cysteine endopeptidase, WP_043038927 (*S. equi* subsp. *equi*) (6), IgG endopeptidase, ABH04315 (*S. equi* subsp. *zooepidemicus*) (7), and IdeS/Mac family cysteine endopeptidase, WP_052123802 (*S. phocae*). (**B**) Dendrogram showing the pattern of relatedness of the aligned sequences, created using Clone Manager Professional 9.

The sequences of known streptococcal IdeS/Mac family cysteine endopeptidases were aligned and analyzed for sequence similarity. This included a sequence from *S. canis* that encodes the protein described here as IdeC. Fig. 1A shows a side-by-side comparison of these proteins; identity scores were generated in Geneious 11.1.5 to describe the similarities of the proteins to each other. IdeS of *S. pyogenes* had the highest similarity to IdeC, with an identity score of 72%. The protein with the second highest similarity to IdeC is the protease of *S. halichoeri*, with a similarity of 71%. The protease from *S. castoreus* was most similar to the proteases of *S. equi* with a score of 60% similarity to both the protease of *S. equi* subsp *zooepidemicus* and *S. equi* subsp *equi*, suggesting a closer relationship between these species. The *S. phocae* protease had the lowest similarity to any of the other proteases with a maximum similarity score of 49% (to IdeC of *S. canis*). The relationships between these proteins have also been demonstrated using a dendrogram to illustrate which proteins are more similar (Fig. 1B).

### Species specificity of IdeC cleavage

**Fig 2:**
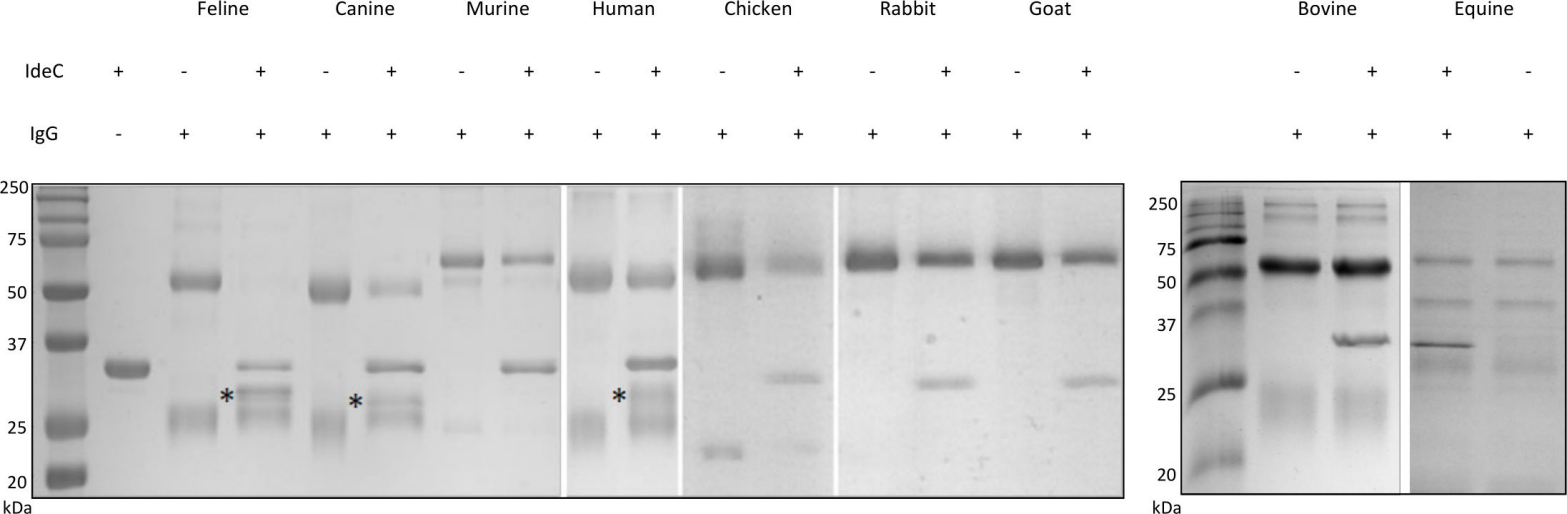
Species specificity of IdeC IgG cleavage. Representative gel images illustrating the ability of IdeC to cleave IgG from various species. Feline, canine, murine, human, chicken, rabbit, goat, bovine, and equine IgG was assessed, and only feline, canine, and human IgG were successfully cleaved by IdeC. The recombinant IdeC has a molecular weight of ~35 kDa; 3 µg each of IgG and IdeC were used in IgG cleavage assays. Reactions were incubated for 3 h at 37°C before being run on a 15% SDS-PAGE gel. Heavy and light chains of IgG are visible at 50 and 25 kDA, respectively, and the cleavage product is visible at 28 kDa and is marked with *.

The IgG cleavage properties of IdeC were investigated by incubating purified recombinant IdeC with purified IgG or serum from different species for 3 h at 37°C and subsequently analyzing the band pattern (Fig. 2). The band patterns were compared with IdeC and IgG incubated independently of each other. IdeC can be seen in Fig. 2 at ~35 kDa. In this study, the effects of IdeC on IgG from cats, dogs, mice, humans, chickens, rabbits, horses, cows, and goats were assessed. In the IgG, only sample protein bands were detected at approximately 50 kDa and 25 kDa, respectively—the heavy and light chains of the immunoglobulin. In the case of the feline, canine, and human IgG incubated with IdeC, faint bands are visible where the heavy chain should be, and a new band is visible at ~28 kDa. The presence of this new band and the diminished strength of the heavy chain led to the conclusion that the heavy chain is cleaved, and the new band is a cleavage fragment. In the case of the mouse, chicken, rabbit, horse, cow, and goat IgG, no cleavage product was observed.

**Fig 3:**
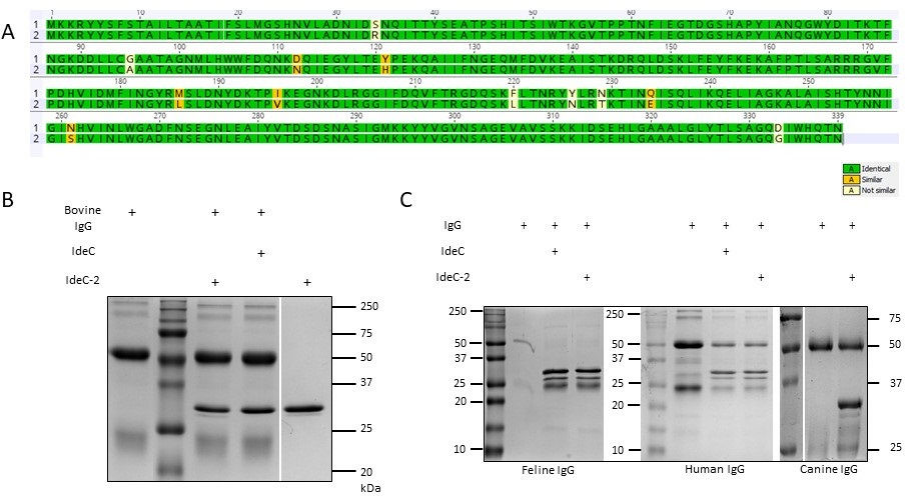
A second form of IdeC was observed in only bovine isolates. (**A**) IdeC-2 (2) has 12 amino acid differences when compared with the wild-type protein (1), and comparison was performed using Geneious 11.1.5. (**B**) IgG cleavage assays were performed to determine whether this protein variant preferentially cleaved bovine IgG. (**C**) IgG cleavage assays demonstrated the cleavage of feline, human, and canine IgG by IdeC-2. For both B and C, 3 µg of IgG and/or recombinant protein was used in the assay. Reactions were incubated for 3 h at 37°C and then run on a 15% SDS-PAGE gel.

During the genetic screening of clinical samples in our collection, we identified a second allele of IdeC that differs by 12 amino acids (Fig. 3A), which was termed IdeC-2. IdeC-2 only appeared in isolates of bovine origin; hence, an additional IgG cleavage assay was performed to assess whether this IdeC type cleaved bovine IgG. However, there was no difference in the cleavage abilities of the IdeC types (Fig. 3C), and neither cleaved bovine IgG (Fig. 3B). These observations taken together demonstrate that IdeC cleaves IgG in a species-specific manner.

### IdeC is a cysteine protease

**Fig 4:**
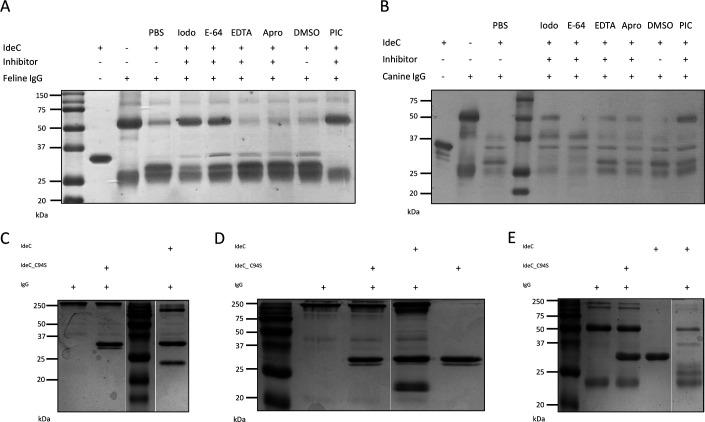
Identification of IdeC as a cysteine protease. After incubation of IdeC (1.5 µg) with different protease inhibitors (2.5 µM) for 30 min at room temperature, 5 µg feline IgG (A) or canine IgG (B) was added, followed by incubation at 37°C for 2.5 h. Protein inhibitors used included protease inhibitor cocktail (PIC), cysteine protease inhibitors iodoacetamide (Iodo), E-64, and serine protease inhibitor aprotinin (Apro). PBS and DMSO were used as negative controls. (C) Feline, (D) canine, and (E) human IgG underwent IgG cleavage assays with IdeC and Ide_C94S to investigate the role of the Cys94 residue in the protein's catalytic function (IgG cleavage was performed for 4 h at 37°C). All reactions were run on 15% SDS-PAGE gels.

As shown in Fig. 1, IdeC displays high similarity to IdeS, which has been described as a non-calcium-dependent cysteine protease (14, 21). In order to test whether IdeC is also in this category of proteases, the IgG cleavage assays were repeated in the presence of various protease inhibitors. Inhibitor experiments were performed with iodoacetamide (Iodo), aprotinin (Apro), E-64, Protease inhibitor cocktail (PIC), and EDTA (Fig. 4A and B). Similar to the PIC, the cysteine protease inhibitors Iodo and E-64 resulted in weaker cleavage product bands compared with serine protease inhibitor Apo or the metalloprotease inhibitor EDTA. This supports the idea of IdeC as a cysteine protease.

Only a single cysteine residue was found in the amino acid sequence of IdeC, at position 94. Using site-directed mutagenesis, an IdeC variant with the hypothetical active site cysteine replaced with a serine residue was produced to assess the role of this residue in the IgG cleaving action of IdeC. Using the IdeC_C94S recombinant protein variant in IgG assays revealed that the Cys94 residue is essential for IgG cleavage by IdeC. Fig. 4C through E shows that for feline, canine, and human IgG, the Csy94Ser substitution results in a loss of IgG cleaving ability by the protein.

### IdeC cleaves between the hinge and CH2 regions of IgG

**Fig 5:**
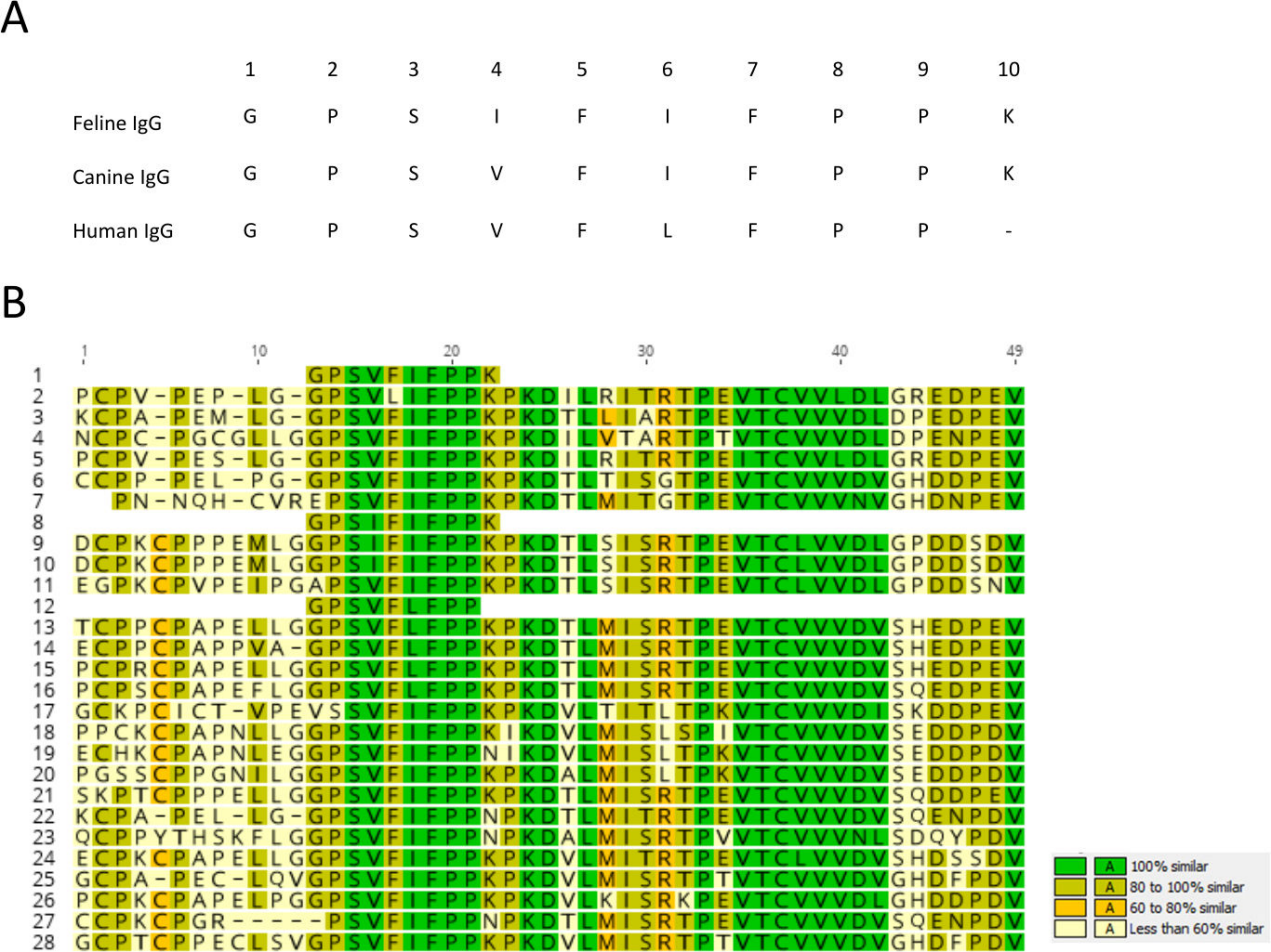
Sequence of the cleavage fragment produced by IdeC cleavage of IgG. (**A**) The cleavage fragments produced during IgG digestion by IdeC were analyzed by N-terminal Edman sequencing, and fragments from feline, canine, and human IgG were analyzed. (**B**) The amino acid sequences of the fragments were aligned to the IgG subtype sequences (subtypes listed here with NCBI accession numbers) from various species using Geneious 11.1.5 (1). Sequence of the canine cleavage fragment (2–5). Sequences of the canine (*Canis lupus familiaris*) IgG isotypes (2), IgG A; AAL35301 (3), IgG B; AAL35302 (4), IgG C; AAL35303 (5), IgG D; AAL35304 (6, 7). Sequences of the bovine (*Bos Taurus*) IgG isotypes (6); IgG 1; ABE68619 (7), and IgG 2; S06611 (8). Sequence of the feline cleavage fragment (9–11). Sequences of feline (*Felis catus*) IgG isotypes (9); IgG 1 a; BAA32229 (10), IgG 1b; BAA32230 (11), and IgG 2; AHH34165 (12). Sequence of the human cleavage fragment (13–16). Sequences of human [*Homo sapiens*] IgG isotypes (13); IgG 1; P01857 (14), IgG 2; P01859 (15), IgG 3; P01860 (16), and IgG 4; P01861 (17–20). Sequences of mouse (*Mus musculus*) IgG isotypes (17); IgG 1; P01868 (18), IgG 2A; P01863 (19), IgG 2B; P01867 (20), and IgG 3; P03987 (21). Sequence rabbit (*Oryctolagus cuniculus*) IgG; P01870 (22–28). Sequences of the equine (*Equus caballus*) IgG isotypes (22); IgG 1; Q95M34 (23), IgG 2; CAC44761 (24), IgG 3; CAC86339 (25), IgG 4; CAC44762 (26), IgG 5; CAC86340 (27), IgG 6; CAC86341 (28), and IgG 7; CAC44763.

The IgG cleavage fragments produced by IdeC were analyzed to determine the cleavage site. The fragments were analyzed by Edman sequencing, and the first 10 amino acids of each fragment were determined. A high degree of sequence similarity was observed when comparing fragments from the canine, feline, and human fragments. The only deviations found were at positions 4 and 6 (Fig. 5A). The feline fragment has an isoleucine at position 4 where the human and canine fragments have a valine; both are hydrophobic, non-polar amino acids. Second, the human fragment has a leucine at position 6, whereas the others have an isoleucine again, both similar residues. The available sequences for the IgG sub-types of all the tested species’ IgG were aligned with the cleavage fragment sequences (Fig. 5B). This showed that the cleavage fragment sequence was found, with very little deviation from the consensus, in the IgG sequences from these species. The similarity of the sequences to the cleavage fragment was between 60% and 100%. A complete match in all IgG was found for the amino acids serine (position 3 in the fragment sequence), phenylalanine (position 7), and proline (positions 8 and 9) (Fig. 5B). Instead of glycine (Gly 1), an arginine residue was found in equine IgG6, a valine residue in murine IgG1, and an alanine residue in feline IgG2. A proline residue was detected at the second position of the cleavage fragment sequence, which was replaced by a serine residue only in murine IgG1. The valine residue at position 4 was found in all species IgG except in the feline cleavage fragment and IgG1a and b, where an isoleucine was detected at this position. All cleavage fragments and all species IgG had a phenylalanine residue at position 5, with the exception of canine IgGB (leucine). Each of the four isotypes of human IgG had a leucine residue at position 6, whereas a phenylalanine residue was always found in the other species. The last position of the fragment sequence was formed by a lysine residue in all IgG except equine IgG1, 2, and 6 and murine IgG2B, which had an asparagine residue at this position. Chicken, donkey, and goat IgG, which were also investigated for host specificity of IdeC, could not be included in the sequence comparison because no amino acid sequences of IgG molecules from these species were available in the NCBI database.

**Fig 6:**
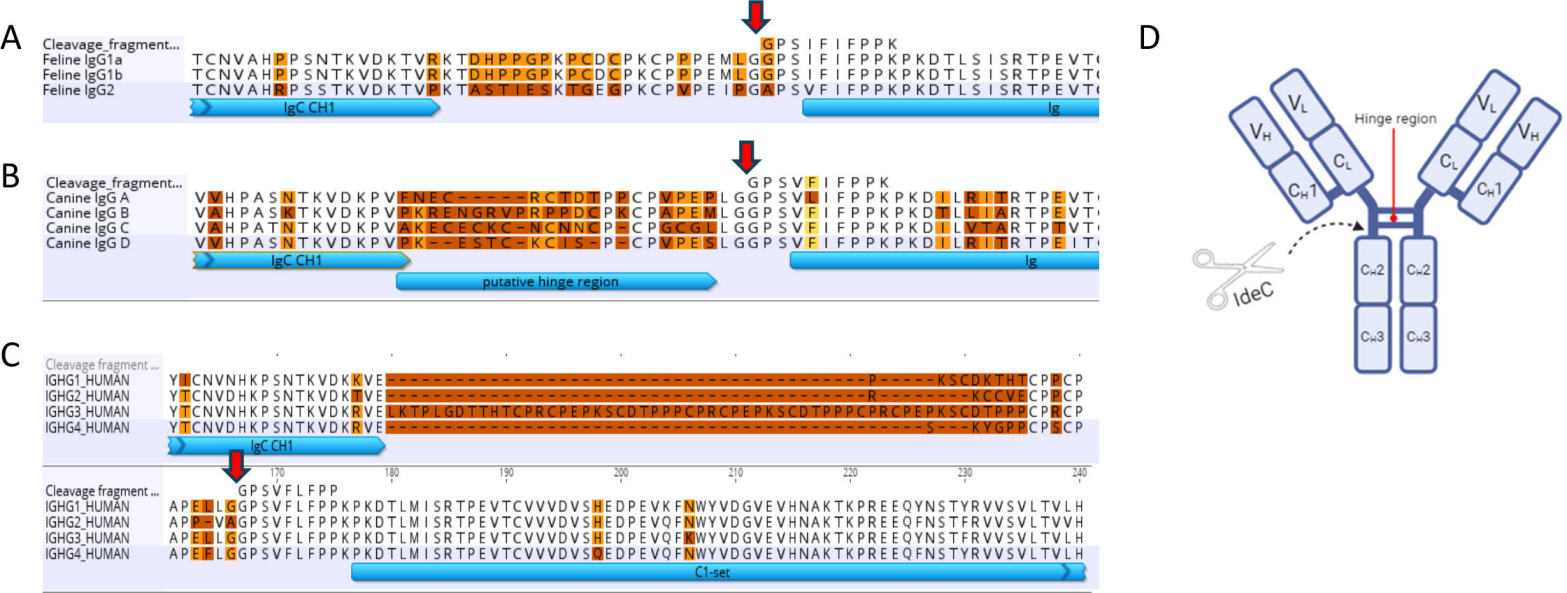
The cleavage site of IdeC is located between the hinge region and the CH_2_ region. (A–C) The feline (**A**), canine (**B**), and human (**C**) cleavage fragments (identified by Edman sequencing) were compared with the IgG subtype sequences from the corresponding species to identify where the cleavage occurs. Geneious 11.1.5 was used to compare sequences. The red arrow indicates the cleavage site. (**D**) Cleavage by IdeC occurs between the hinge region and the CH2 region of IgG.

To identify the IdeC cleavage site in the IgG molecules, the sequences of the cleavage fragments were aligned to the sequences of canine, feline, and human IgG (Fig. 6). The sequences for canine and feline IgG isotypes were taken from the NCBI protein database (Accession numbers; *Canis lupus familiaris* IgGA: AAL35301, IgGB: AAL35302, IgGC: AAL35303, IgGD: AAL35304, *Felis catus* IgG1a: BAA32229, IgG1b, IgG2: AHH34165). Only one deviation in the sequence was found in the canine sub-types. In IgG A, at position 5 of the cleavage fragment, phenylalanine was replaced with leucine in the heavy chain of IgGA. In the alignment of the feline IgG sub-types, two differences were present in IgG2; at the first position, a glycine was replaced with an alanine, and at the fourth position, an isoleucine was replaced with a valine. The sequences for the human sub-types were taken from UniProt (*Homo sapiens* IgG1: P01857, IgG2: P01859, IgG3: P01860, and IgG4:P01861). There were no amino acids changed from the cleavage fragment to any of the human sub-types of IgG. This alignment showed the cleavage fragment was located between the hinge region and the CH2 region in all IgG sub-types.

### Binding efficiency of IdeC

**Fig 7:**
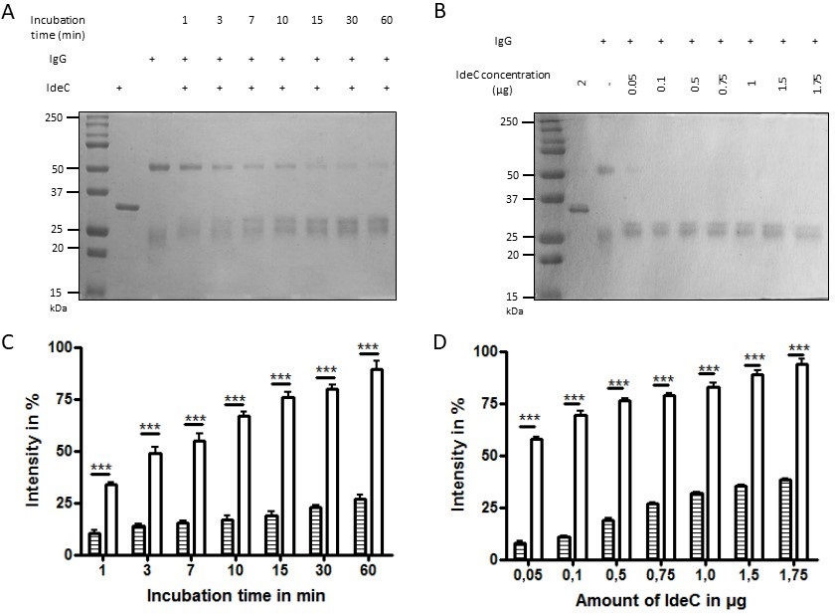
Cleavage efficiency of canine and feline IgG by IdeC. (**A**) Representative image of the gel showing feline IgG cleavage by IdeC; reactions were performed with incubation times varying between 1 and 60 min; 2 µg of both IgG and IdeC was used. (**B**) Representative gel images showing digestion of feline IgG by varying concentrations of IdeC (0.05–1.75 µg) and 2 µg of IgG were used, and each reaction was incubated for 3 h. For both A and B, 15% SDS gels were used. (C & D) The cleavage efficiency of feline (lined bars) and canine (black bars) IgG when (**C**) incubation time is varied, incubation times between 1 and 60 min were used, or (**D**) IdeC concentration is varied, and concentrations used were between 0.05 and 1.75 µg. Analysis was performed three times, and percentages were generated by analyzing all gel images using GelQuant.NET. The bands representing the digested heavy chain (at 50 kDA) were compared with the undigested heavy chain to determine the percentage of heavy chain digested. Significance was calculated using a student’s *t*-test. *** indicates a *P* value < 0.001.

To take a closer look at the interaction of IdeC with feline and canine IgG, cleavage assays were repeated with variable IdeC concentration or incubation times. The intensity of the cleavage fragment was quantified and compared between feline and canine IgG. As the concentration of IdeC or incubation time increases, so does the level of cleavage observed for both feline and canine IgG (Fig. 7A and B). However, in all experiments, the level of cleavage of feline IgG is significantly higher than the cleavage of canine IgG.

**Fig 8:**
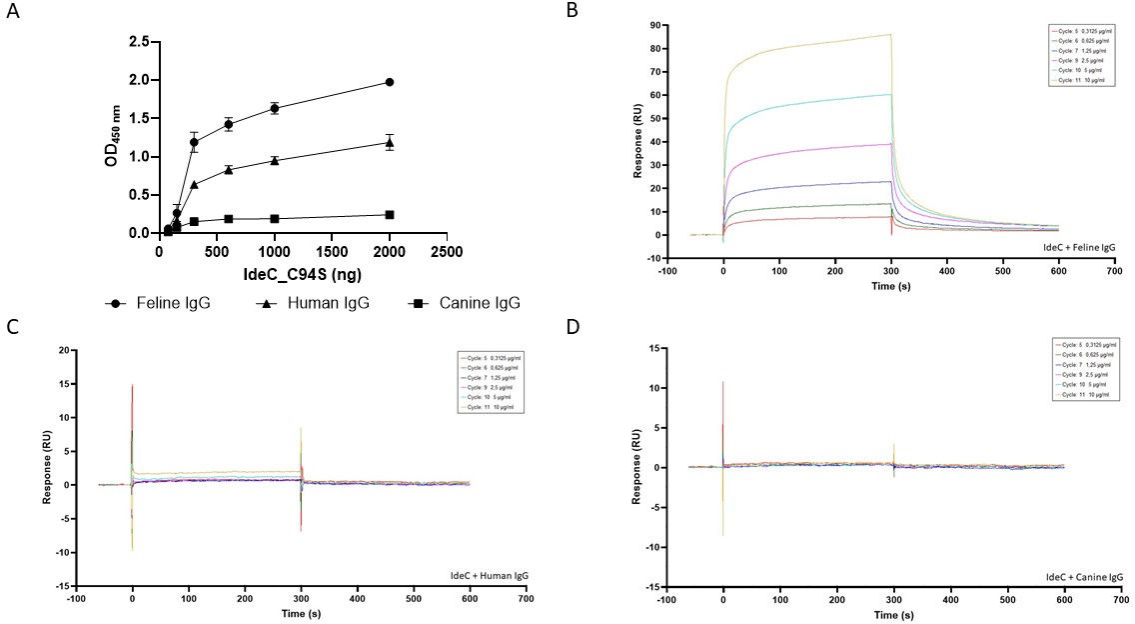
IdeC shows the greatest binding affinity for feline IgG. (**A**) The ELISA assay shows greatest binding occurs with feline IgG, followed by human IgG, and finally, canine IgG shows the least binding affinity. In a 96-well plate, wells were coated with increasing amounts of IdeC_C94S (75–2,000 ng). The interaction of HRP-conjugated feline, canine, and human IgG was measured by taking optical density readings at OD_450nm_. (**B-D**) Biacore analysis of the interaction between IdeC_C94S and feline (**B**), canine (**C**), and human (**D**) IgG. Binding was measured using a BIAc BIAcore T200cal biosensor; various IgGs were immobilized as ligands on the sensor chip. Binding analysis was performed with IdeC_ C94S added at concentrations ranging from 0.3125 to 10 µg/mL in PBS 0.05% Tween R-20 at 25°C using a flow rate of 10 µL/min, and all interactions were measured in triplicate. Panels B–D show control corrected sensorgrams obtained by subtraction of data measured from a control flow cell with no immobilized protein, referred to here as blank subtracted. Colored lines represent measurements of binding corresponding to different concentrations of IdeC; 0.3125 (red), 0.625 (green), 1.25 (indigo), 2.5 (magenta), 5 (cyan), and 10 µg/mL (gold).

The binding of IdeC to canine, feline, and human IgG was further assessed using an ELISA assay with wells coated with increasing concentrations of IdeC_C94S. As seen in Fig. 8A, the binding of IdeC and feline IgG was the strongest of the three tested species IgG, followed by human IgG, and finally, the lowest degree of binding was seen between IdeC and canine IgG. For both feline and human IgG, the strength of binding increased as the concentration of IdeC increased, across the range of 75–2,000 ng. However, canine IgG showed no increasing interaction with IdeC with increased concentration. The ELISA data are supported by the Biacore analysis, where significant interaction of IdeC was only observed with feline IgG. Even the lowest concentration of IdeC_C94S measured, 0.3125 µg/mL, shows binding to feline IgG over time (Fig. 8B). This interaction increases in strength with increased concentration of IdeC_C94S, agreeing with the ELISA data (Fig. 8A and B). The interaction of canine IgG with IdeC is also similar in both ELISA and Biacore analyses; in both Fig. 8A and D, little to no binding ability of IdeC_C94S to canine IgG is demonstrated. IdeC binding of human IgG, as measured by Biacore analysis (Fig. 8C), showed only a very low level of interaction between IgG and IdeC at the two highest concentrations of IdeC_C94S (5 and 10 µg/mL).

### Deglycosylation of feline IgG affects cleavage

**Fig 9:**
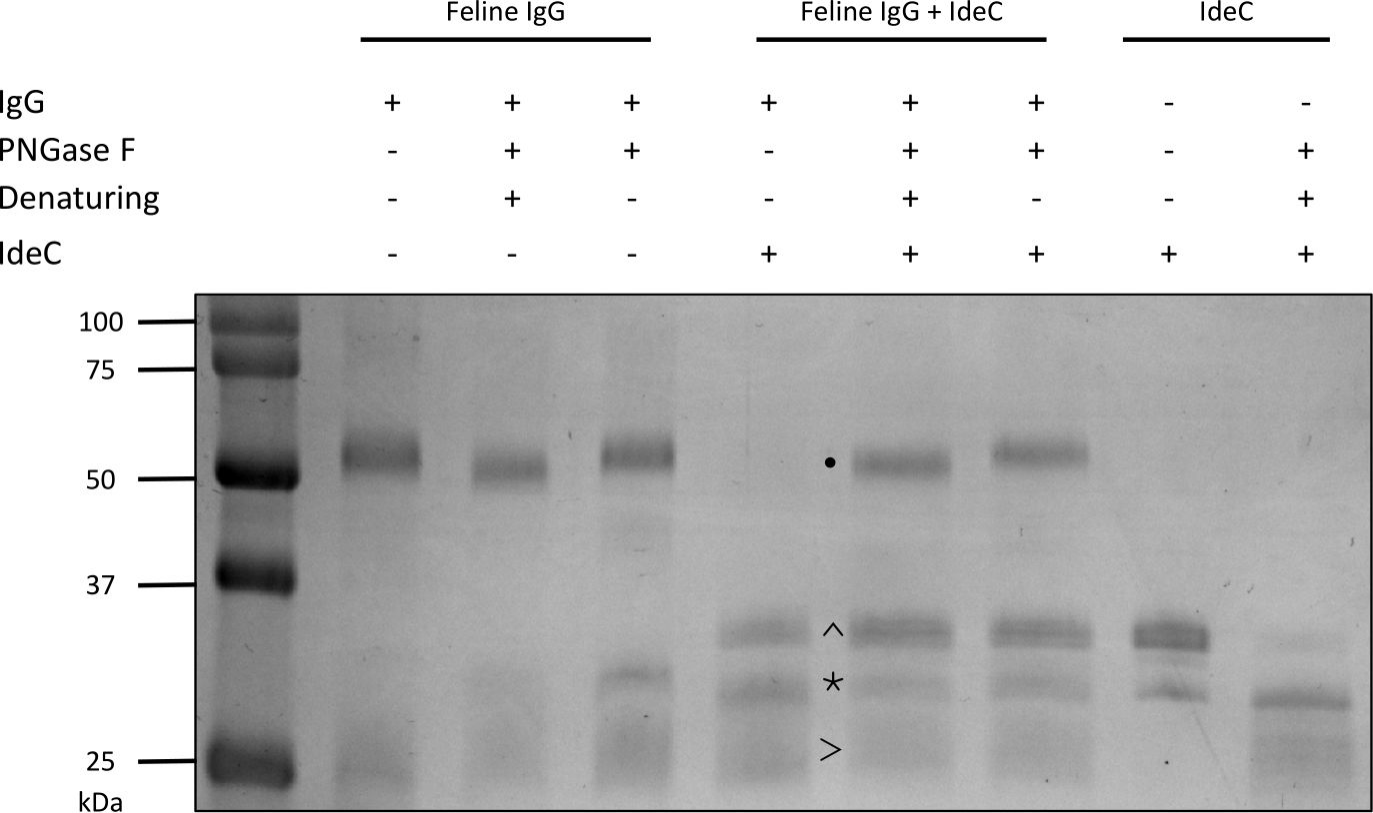
Deglycosylation of feline IgG has an impact on cleavage. *N-*glycosylation supports the cleavage of feline IgG; 2 µg of IgG and/or IdeC were used, and 1 µL of PNGase F was used to remove N-glycans. Reactions were incubated for 3 h at 37°C and run on a 15% SDS-PAGE gel. The heavy chain of IgG is marked by •, the light chain of IgG is marked by >, IdeC is marked by *, and the cleavage fragment is marked by ^.

*N*-glycosylation is a common regulatory post-translational modification of immunoglobulins; most commonly, this entails the glycosylation of asparagine residues in the heavy chain of IgG (10). Canine and feline IgG were subjected to deglycosylation by incubation with PNGase F prior to cleavage assays to assess the relevance of glycosylation in IgG cleavage by IdeC. We determined that *N*-glycosylation of feline IgG supports cleavage (Fig. 9); it did not have an effect in the case of canine IgG cleavage (Fig. S1).

### Three-dimensional structure of IdeC

**Fig 10:**
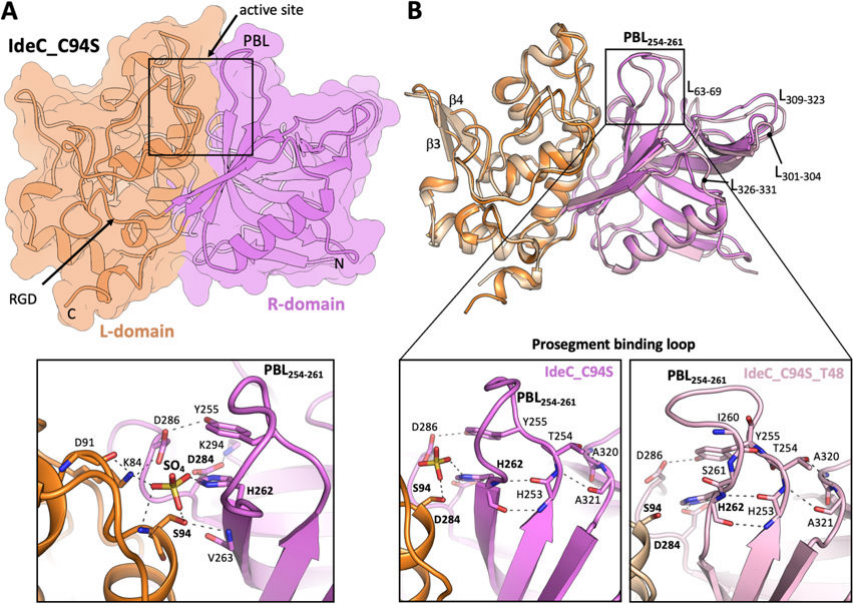
Three-dimensional structure of IdeC. (**A**) The overall structure of IdeC_C94S is shown in both surface and cartoon representations, with domains highlighted in different colors: the L-domain in orange and the R-domain in pink. Arrows indicate the active site and the RGD motif. A close-up view of the IdeC active site is depicted below, with a sulfate ion and conserved residues represented as sticks. Hydrogen-bond interactions are illustrated with dashed lines. (**B**) Structural comparison between the crystallographic structures of IdeC_C94S (dark orange and pink) and IdeC_C94S_T48 (light orange and pink). Labeled regions highlight structural differences. Bottom frames display zoomed-in views of the prosegment binding loop (PBL) in both IdeC_C94S and IdeC_C94S_T48 structures.

In an attempt to better understand the structure and function of IdeC, the crystal structure of IdeC (residues 30–339), with a C94S mutation to abolish catalytic activity, was solved at 2.7 Å resolution (Table 1 and Fig. 10A). The crystals belong to the P3_2_ 2 1 space group with one molecule per asymmetric unit. The electron density map allowed perfect modeling of residues 48–339, whereas no electron density was visible for either the N-terminal residues 30–47 or the polyhistidine tag, indicating an inherent flexibility at the N-terminal end of the protein. The structure of IdeC presents the α/β fold characteristic of the papain superfamily of cysteine proteases, consisting of an α-helix followed by an antiparallel β-sheet comprised of four/five β-strands (22). Among the highest structural homologs to IdeC, according to Foldseek (23), is the *S. pyogenes* cysteine protease IdeS/Mac-1 (PDB:1Y08, rmsd of 0.393 247 Ca atoms and PDB: 2AVW, and rmsd of 0.448 239 Ca atoms) (21, 24).

**TABLE 1:** Crystallographic data collection and refinement statistics^*a*^

Data collection	IdeC_C94S	IdeC_C94S_T48
Space group	*P* 3_2_ 2 1	*P* 2_1_ 3
Cell dimensions		
*a, b, c (Å)*	70.25 70.25 94.88	109.38 109.38 109.38
*α, β, γ (°)*	90, 90, 120	90, 90, 90
Wavelength (Å)	0.979	0.979
Resolution (Å)	47.44-2.70 (2.83-2.70)	48.92-2.25 (2.32-2.25)
*R_pim_*	0.138 (0.792)	0.059 (0.706)
CC_1/2_	0.977 (0.447)	0.997 (0.431)
Mean *I/σI*	4.8 (1.0)	11.3 (1.2)
Multiplicity	9.5 (9.5)	12.6 (12.7)
No. unique reflections	7,819 (1,006)	21,011 (1,916)
Completeness (%)	99.9 (100)	100 (100)
**Refinement**		
Resolution (Å)	37.44-2.70 (2.77-2.70)	48.96-2.25 (2.31-2.25)
*R_work_/R_free_*	0.2017/0.2675	0.1782/0.2137
No. of atoms		
Non-hydrogen atoms	2,368	2,457
Protein	2,311	2,297
Ligands	25	0
Solvent	32	160
Ramachandran favored (%)	94.00	96.00
Ramachandran allowed (%)	6.00	4.00
Ramachandran outliers (%)	0.00	0.00
Average B, all atoms	50.0	42.0
RMSD		
Bond lengths (Å)	0.004	0.009
Bond angles (°)	1.080	1.606
PDB entry	9HB1	9HB2

^*a*^ Values in the parentheses correspond to the higher resolution shell.

**Fig 11:**
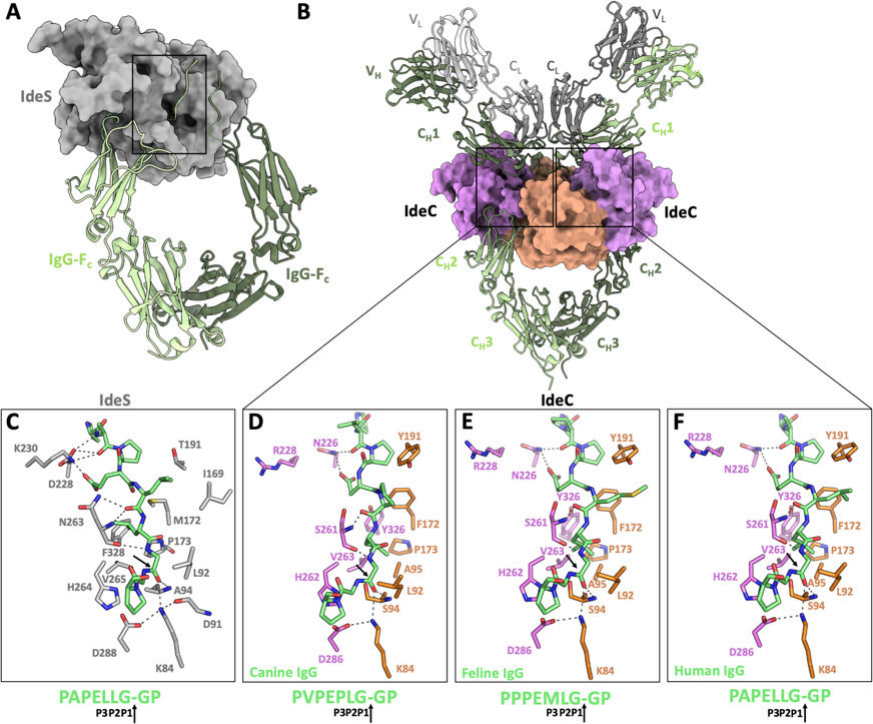
Predicted IgG substrate binding by IdeC. (**A**) Crystallographic structure of the related *S. pyogenes* IdeS protein (represented as a gray surface) in complex with IgG-Fc, depicted in the cartoon of various shades of green (PDB:8A47). (**B**) AF model of the IdeC:IgG A canine model. IdeC is represented on the surface, colored by the domain (orange for the L-domain and pink for the R-domain). The immunoglobulin heavy chains are represented in various shades of green, and the light chains are represented in different grays. (**C-F**) Detailed views of substrate recognition. (**C**) IgG hinge recognition by IdeS active site in PDB:8A47. (**D**) Model of canine IgGA recognition by IdeC. (**E**) Model of feline IgG1a recognition by IdeC. (**F**) Model of human IgG1 recognition by IdeC.

IdeC chain contains two globular domains, the L- and R-domains, which correspond to the left and right orientations in the standard view for papain (Fig. 10A). The active site, the canonical catalytic triad for papain-like cysteine peptidases (Cys-His-Asn/Gln/Asp/Glu), is situated at the interface and involves residues from both domains (Fig. 10A). The catalytic cysteine, Cys94, mutated to a serine in the structure, is located in the L-domain, whereas the catalytic histidine, His262, is found in the R-domain (Fig. 10A: bottom). Asp284 (also in the R-domain) completes the catalytic triad by forming hydrogen bond interactions with His262, orienting its imidazolium ring for the proteolytic reaction. Additionally, Asp284 forms a salt bridge with Lys294 (Fig. 10A). The oxyanion hole is formed by Lys84 and the peptide amide of the catalytic Cys94. The position of the side chain of Lys84 is secured by a network of interactions (the salt bridge between Lys84 with Asp286, and H-bond interaction between Asp286 and Tyr255) that shield the catalytic residues as observed in the Mac-1 cysteine protease of *S. pyogenes* (24). Interestingly, a sulfate ion was present at the active site in the structure, with one of its oxygen hydrogen-bonded to the N^z^ of Lys84 and the amide of Ser94, mimicking the interaction of the scissile carbonyl group of the substrate and the oxyanion hole (Fig. 11A: bottom), which was also observed in other related members of the same family (24). The remaining oxygens of the sulphate ion establish hydrogen bonds with His262 and the side- hain of Ser94 (Fig. 11A: bottom).

Residues 254–261 of the IdeC structure form a loop that is equivalent to the prosegment binding loop (PBL) found in other papain superfamily cysteine proteases (Fig. 10A). The PBL in IdeC, interestingly only eight residues long (in other members of the papain superfamily being around 17–18 residues long (24)), partially obstructs substrate binding and is structured by hydrogen-bond interactions involving the main chains of His253, His262, Ala320, and Ala321 and the side chains of Thr254, Tyr255, His262, Asp284, and Asp286 (Fig. 10B). Additionally, residues 214–216 contain an RGD motif. In the IdeC structure, this motif is solvent-exposed, situated in the L-domain, and is positioned away from the active site (Fig. 10A).

To enhance crystal quality, a truncated form of the inactive IdeC was produced in which the first 48 residues were removed (IdeC_T48_C94S). Crystallization experiments with the truncated IdeC produced new crystals (Table 1) in the cubic *P* 2_1_ 3 space group that allowed the structural determination of this variant at 2.25 Å resolution (Fig. 10B and Fig. S2). Comparison with the IdeC_C94S structure revealed a high degree of similarity (rmsd of 0.376 Å for 229 Ca atoms superimposition), although notable differences were located at the PBL loop, further closing the cavity and blocking the substrate binding site in the IdeC_C94S_T48 structure (Fig. 10B). No sulfate ion was found in the IdeC_C94S_T48 active site, which may account for the different conformations of the PBL. In the IdeC_C94S_T48 structure, the PBL is stabilized by polar interactions involving the main chains of His253, Tyr255, Ile260, His262, Ala320, and Ala321, as well as with the side chains of Thr254, Tyr244, and His262 (Fig. 10B). Additional structural differences between both structures were noted in loops 63–69, 186–194, 226–231, 301–304, and 309–323 and in residues 125–137, which form a small β-sheet (β−3 and β−4) in the truncated IdeC structure. Minor changes were observed between residues 86–89, 160–163, and in α−2 (Fig. 10B).

### Structural modeling of the recognition of feline/canine/human IgGs by IdeC

Immunoglobulin substrate recognition by IdeS was recently characterized by X-ray crystallography (Fig. 11A) (25). Similarly, we conducted crystallographic experiments with the truncated form of IdeC and the IgG-Fc. Unfortunately, only crystals of IdeC alone were obtained. Modeling studies of the IdeC:IgG complex were carried out using Alphafold 3 (26), which provided a reliable model for IdeC in complex with the full-length canine IgGA (see Methods, Fig. 11B and Fig. S3A). Although different oligomeric states are known for cysteine proteases—IdeS/Mac 1 has been reported to function both as a monomer (25, 27) and dimer for immunoglobulin recognition (24)—the monomeric state was the most abundant in IdeC (Fig. S4), and the only one observed in our crystal structures. In the same sense, AF3 does not provide any reliable model for the IdeC dimer. Thus, two independent IdeC molecules were included in the complex modeling, as two separate cleavage sites for IdeC are available on an IgG molecule. The AF3 model presents two monomers of the IdeC attached to the full IgG in a Y-shaped configuration (Fig. 11B), with the predicted IdeC model being nearly identical to the crystallographic structure (rmsd of 0.366 Å 250 Cα atom superimposition) (Fig. S3B and S3C). In this model, each IgG Fc hinge peptide was positioned within the active site of one IdeC molecule (colored by domains as in Fig. 10), with the two IdeC molecules located on opposite sides of the immunoglobulin (Fig. 11B). Thus, our results indicate that two IdeC chains could act on IgG simultaneously without steric impediments, but it is also equally feasible that a single IdeC chain attacks IgG in two consecutive steps.

To explore in depth the structural determinants defining the specificity of IdeC, the crystallographic IdeS:IgG-Fc structure (24) was compared with our IdeC:canine IgGA model. Additionally, the IdeC substrate residues (P2′-P7), identified in this study for the *Felis catus* IgG1a and the *Homo sapiens* IgG1, were also modeled into the IdeC active (Fig. 11). In both the IdeS:IgG-Fc complex and the IdeC:IgG models, the IgG hinge region is primarily stabilized in the cavity by polar contacts (Fig. 11C through F). The carbonyl oxygen of the scissile bond (Gly-P1) (indicated by an arrow in Fig. 11C through F) is anchored in the oxyanion hole, forming hydrogen bonds with the peptide amide of Cys94 and the side chain of Lys84. This orientation positions the amide nitrogen of the P1 residue close to the imidazole ring of His262. In all cases (IdeS and IdeC complexes), the catalytic site presents a very narrow cavity allowing only the entrance of Gly or Ala residues at positions P1 and P1’. The adjacent position (P2) is occupied by leucine in all three substrates modeled (Fig. 11D through F). As detailed below, the models for IdeC:IgG complexes explain the preferential activity of IdeC against feline IgG. Position 3 (P3) for the feline IgG presents a bulky hydrophobic residue (Met) that is nicely accommodated in a hydrophobic pocket built by Phe172, Pro173, and Tyr191 (Fig. 11E). Although P3 in human IgG presents a Leu residue and canine IgG a Pro residue (Fig. 11F and D), explaining the reduced activity for canine IgG versus feline and human IgG.

## DISCUSSION

*S. canis* is a multi-host pathogen with a broad range of clinical presentations (3, 5). However, there is still limited knowledge relating to the virulence determinants of this bacterium, excepting the M-like protein; SCM (28, 29). Streptococcal proteases have been characterized for many important streptococcal pathogens. This study describes the IgG cleavage properties of IdeC, an IdeS/Mac-like protease.

The protease type of an enzyme is determined based on the amino acids that make up the catalytic center of the protein. Based on the high sequence similarity to IdeS (14, 21), IdeC was predicted to be a cysteine protease (Fig. 1). The 3D structure of the protein (Fig. 10) revealed, again, similarities to IdeS of *S. pyogenes*, with the predicted catalytic triad being located at the center of the two domains of the protein, where the substrate interacts. This assumption can be investigated with the use of protease inhibitors. Pre-incubation of IdeC with cysteine protease inhibitors E-64 and iodoacetamide before IgG cleavage assays suggested IdeC was indeed a cysteine protease rather than a serine protease. Inhibition of IdeC’s cleavage ability was more pronounced in canine IgG, particularly when pre-incubation with E-64 had been performed (Fig. 4A and B). Iodoacetamide irreversibly inhibits cysteine proteases by alkylation of the catalytic cysteine in the active site (30), and E-64 binds to the subunits of cysteine proteases, and irreversible inhibition occurs (31). For IgG-specific proteases from other streptococci, such as IdeS, IgdE, IdeP, and the IgM-specific protease IdeS_suis_ from *S. suis*, it has already been described that they are also inhibited by iodoacetamide, but not by E-64 (15, 16, 21, 32). The lack of inhibition by this classic cysteine protease inhibitor in other streptococcal proteases may be due to differences in the active site cleft of these proteins compared with other papain-like cysteine proteases (21). The fact that E-64 did show some inhibitory effects on IdeC suggests possible differences in the catalytic activity of the protein despite high sequence similarity to IdeS. Indeed, our models of the proteins’ interactions with IgG (Fig. 11A and B) suggest that IdeS and IdeC interact with immunoglobulin according to different mechanisms. Furthermore, mutation of IdeC to replace the Cys94 residue with serine, removed all IgG cleaving properties of IdeC (Fig. 4C through E), confirming that this residue is vital for the interaction as it is in IdeS (33).

As *S. canis* is a multi-host pathogen, the ability of IdeC to cleave IgG from various species was assessed. Recombinant IdeC cleaved feline, canine, and human IgG, but IgG from mice, chickens, rabbits, horses, goats, and cows was not cleaved (Fig. 2). As mucosal colonization and infections have been reported frequently in dogs and cats and more rarely in humans, it is logical that IdeC is capable of the cleavage of these species’ IgG. However, it is more surprising that bovine IgG is not cleaved, as cows are also the common hosts of *S. canis*. Other streptococcal cysteine proteases have been demonstrated to interact specifically with the IgG of their associated host. For example, IgdE, isolated from *S. suis*, cleaves porcine IgG, but not human, goat, cow, horse, or mouse IgG (16). Similarly, IdeP of *S. phocae* has been demonstrated to cleave IgG from gray seals and sea dogs, but not of other marine mammals (15). There also appear to be differences in the effectiveness of cleavage between human, feline, and canine IgG, based on the level of degradation of the heavy chain; feline IgG is the most successfully digested, followed by canine and then human IgG (Fig. 2). However, the mechanism of host specificity remains unclear.

As stated above, although *S. canis* is a common causal factor in bovine mastitis (1, 34, 35), no cleavage of bovine IgG occurs via IdeC. During genome analysis of clinical isolates, we observed the presence of a possible second form of IdeC that was associated with bovine strains. In a previous study, Richards et al. reported that bovine and canine strains were significantly different (35). We repeated the IgG cleavage assays with the purified IdeC found in bovine isolates to assess whether the bovine isolates of *S. canis* had an IdeC subtype that cleaved bovine IgG. This did not appear to be the case. As one of the main hosts of *S. canis*, it seems strange that no cleavage occurs of bovine IgG. However, it is possible that a second IgG protease is present in the *S. canis* genome that interacts with bovine IgG. There are other streptococcal species with multiple IgG proteases reported, for example, *S. pyogenes* and *S. equi* subsp. *equi* (14, 18, 30). Another possible explanation for the lack of IgG cleavage in bovine hosts could be the difference in disease presentation; among bovine isolates, mastitis is the most reported infection, whereas in both felines and canines’ skin and mucosal infections are more prevalent (5). Possibly, IgG cleavage is not as vital in this environment, although IgG is the most prevalent immunoglobulin in cow’s milk (36). More likely, *S. canis* is not adapted to bovine hosts. Supporting this theory is the fact that, though it can cause mastitis, *S. canis* has not been reported to colonize the udder.

To try and understand the species specificity of the IgG cleaving ability of IdeC as well as what effect IgG subtypes may have on the results we observed, the sequences for the IgG subtypes of the species tested were compared with the sequences of the cleavage fragment produced by IdeC action. However, the comparison of the amino acid sequences of the IgG of other species provided no evidence for the demonstrated host specificity of IdeC for canine, feline, and human IgG (Fig. 5). The cleavage product fragment was present in all sequence analyses, with minimal variation in amino acids. Fig. 6 provides more insight into the location of cleavage, showing that IdeC acts between the hinge region and the CH2 region. This cleavage site is similar to the cleavage site described for papain and IdeS (37, 38). This cleavage site in the heavy chain of IgG is also supported by the reduction of heavy chain present after cleavage has occurred, compared with the unchanged amount of light chain IgG (Fig. 2).

As discussed, IdeC is an IgG protease; however, there are subtypes of IgG present in humans, canines, and felines (10, 39, 40). In humans, IgG1, IgG2, IgG3, and IgG4 are described, each with a different constant region and biological function (41). IgG1 and IgG3 typically trigger effector mechanisms; in contrast, IgG2 and IgG4 are involved in the induction of more subtle responses (42). Dogs too have four sub-types: IgGA, IgGB, IgGC, and IgGD subtypes (40). Feline IgG subtypes differ slightly with IgG1a, IgG1b, and IgG2 being reported (43). These subtypes are present at different levels in the body and present in unknown ratios in purified IgG preparations. It is demonstrated in Fig. S5 that certain subtypes are more effectively cleaved, in the case of human subtypes. Therefore, it is possible that the composition of the purified IgG sample could result in more or less advantageous ratios of subtypes for cleavage.

Despite having the same cleavage site and high sequence similarity, there were differences in the effectiveness of IdeC cleavage in cats, dogs, and humans. The content of the resulting feline IgG cleavage fragment was always higher than that of the canine fragment for each of the IdeC protein amounts used (Fig. 2). The situation was similar when time-dependent cleavage was compared. Feline IgG was cleaved by IdeC much more effectively than canine IgG even after a short incubation period (Fig. 7B). Additionally, ELISA assays showed feline IgG had a greater binding affinity for IdeC, followed by human and finally canine IgG (Fig. 8). Biacore analysis also indicated feline IgG displayed the strongest binding with IdeC, with human and canine IgG having negligible binding with IdeC (Fig. 8B through D). A possible avenue to explore in elucidating the differential cleavage of canine, feline, and human IgG by IdeC is post-translational modifications. Glycosylation patterns have been shown to be vital for immunoglobulin stability as well as binding (10). Most relevant here, glycosylation has been implicated in increasing resistance to proteases, for example, papain (44). Human IgG is known to have a conserved *N*-glycosylation site in the CH2 domain of both heavy chains of the Fc region (45), very close to our cleavage site. A comprehensive analysis of species-specific glycosylation patterns was performed by Raju et al., and detailed differences in glycosylation across a range of IgG species (46). This study showed that although different species’ IgG may have a high sequence identity, they display differing glycosylation patterns. For example, differences in the structure of the *N*-glycans at the Fc region of the IgG may be clues as to why IdeC is more effective at cleaving feline IgG. Fig. 9 shows that glycosylation is indeed playing a role in the cleavage of feline IgG, with reduced cleavage occurring in deglycosylated IgG, whereas no difference was observed in canine cleavage after deglycosylation. Altogether, our results consistently show that IdeC has a greater binding affinity for feline IgG compared with canine IgG (Fig. 7 and 8). It may therefore be possible that, although first reported in dogs, *S. canis* is more adapted to a feline host and cats may have been the predominant ancestral host.

Analysis of the crystal structure of IdeC, along with modeling of the protein’s interaction with IgG, can also provide insight into the potential reason behind the higher cleavage activity against feline IgG. The crystal structure (Fig. 10) supports the inclusion of IdeC in the papain-like cysteine peptidase superfamily (Interpro IPR038765), although unlike other papain superfamily members, which are typically secreted as zymogens due to the presence of a prosegment N-terminal motif, it is predicted to be secreted as a mature protein (47). Superimposition of the IdeC structure with the IdeS:IgG-Fc complex (PDB: 8A47) (25) reveals a perfect correlation of the sulfate oxygen in the IdeC_C94S structure with the IgG carbonyl oxygen (Gly236) in the IdeS:IgG-Fc complex (Fig. S6). Modeling of the IdeC-IgG complex using AlphaFold 3 gives insight into the difference in interaction with IgGs of different species. The predicted S2 pocket of IdeC is formed by non-polar amino acids, such as Ala95, Pro173, and Val263, which can accommodate Leu, Val, Ala, or Pro (Fig. 5). The three substrates differ in the residue found at the P3 position: canine IgGA has Pro, Met, Leu, or Ser at this position, feline IgG1a has either Met or Ile, and human IgG1 has either Leu or Pro. The IdeC pocket at this position is flexible, allowing it to accommodate nonpolar amino acids of varying sizes. It is possible that variations in stabilization at this position contribute to the different enzyme efficiencies across species. Canine IgGs contain smaller residues at this position, whereas the presence of bulkier residues, such as methionine or isoleucine in feline IgG, may enhance substrate stabilization, leading to the highest cleavage observed in this study.

In this study, we describe the novel IdeS/Mac-like protease IdeC and detail its IgG cleavage capabilities. We demonstrated IdeC is a cysteine protease that cleaves feline, canine, and human IgG between the hinge and CH2 regions, defining a new virulence determinant for *S. canis*. Although *S. canis* is most commonly associated with canine hosts, we observed a more effective cleavage of feline IgG. The sequence of the cleavage site was found to be highly similar in cats, dogs, and humans; therefore, differences in cleavage must not be attributed to sequence differences. Post-translational modifications may play a role instead, for example, differing glycosylation patterns could result in varying degrees of cleavage occurring. Additionally, modeling the interaction of IdeC with the different species’ IgGs indicates that differences in substrate stabilization may contribute to this increased affinity for feline IgG.

## MATERIALS AND METHODS

### Bacterial strains

The *S. canis* strain G361 is a clinical isolate from a human vaginal swab (48), and the *ideC* gene used in this study for protein studies was amplified from this strain. The bovine strain IMT801, kindly provided by Dr. Thomas Peters at the MBFG, was used to amplify the *ideC-2* gene variant. All streptococci were grown in brain heart infusion (BHI) media or on Columbia blood agar without antibiotic selection at 37°C. *E. coli* M15 pREP cells were grown in lysogeny broth (LB) 25 µg/mL kanamycin. M15 pREP cells transformed with the pQE30 plasmid, or constructs made from this plasmid, were grown in LB with 25 µg/mL kanamycin and 100 µg/mL ampicillin.

### Analysis of genetic sequences

Sequence comparison of IdeC to other streptococcal proteases was performed using Geneious 11.1.5. The sequences for the cysteine proteases were taken from the NCBI database, and accession numbers are listed below (Table 2).

**TABLE 2:** The NCBI accession numbers for streptococcal cysteine proteases used in this study

Species	Accession number
*Streptococcus canis*	WP_003044545
*Streptococcus pyogenes*	WP_032462089
*Streptococcus phocae*	WP_052123802
*Streptococcus castoreus*	WP_051188344
*Streptococcus equi* subsp*. Zooepidemicus*	ABH04315
*Streptococcus equi* subsp*. Equi*	WP_043038927
*Streptococcus halichoeri*	WP_159797190.1

### Purification of IdeC-recombinant proteins

Two recombinant IdeC proteins were purified for analysis in this study: G361 and IMT801. The *ideC* gene was amplified from the target strain by PCR using the primers: IdeC-sigseq BamH1 (5′-GCTGGATCCGACAACATCGA-3′) and IdeC HindIII (5′-GGGAAGCTTTTAGTTTGATGCC-3′). This PCR product and the plasmid pQE30 were purified (QIAGEN QIAquick PCR Purification Kit and QIAGEN QIAprep Spin Miniprep Kit, respectively) and digested with BamHI and HindIII (Promega). The digested vector and insert were ligated using Promega T4 DNA ligase. The ligated construct was transformed into M15 pREP cells. To produce the recombinant IdeC_C94S protein, the pQE30_*ideC* construct carrying the G361 *ideC* gene was used in an inverse PCR with the primers IdeCCysSerlong_f (5′-ATGACCTTCTTAGTGCGGCGGCTACCGCAGGAAATATGCT-3′) and IdeCCysSerlong_r (5′-AGCATATTTCCTGCGGTAGCCGCCGCACTAAGAAGGTCAT-3′). The PCR product was purified and ligated with T4 DNA ligase. The pQE30_*ideC_C94S* vector was transformed into M15 pREP. To express the recombinant proteins, the M15 pREP strains were grown at 37°C and 200 rpm to an OD_600_ of ~0.5. 1M IPTG was added, and the culture was grown in the same conditions for 4 h. Cells were collected by centrifugation and lysed using a 1 mg/mL lysozyme solution and then purified by nickel chromatography using Protino Ni-TED 1000 columns according to manufacturer’s instructions. Thermo Scientific Slide-A-Lyzer Dialysis Cassettes 10,000 MWCO were used to exchange the storage media, and proteins were run on a 15% SDS gel to confirm whether purification was successful.

### IgG cleavage assay

Purified recombinant proteins, described above, were used in this assay; 3 µg of the purified IdeC were incubated with 3 µg purified IgG or serum and incubated for 3 h at 37°C, and 4× Laemmli buffer was added to samples and they were denatured at 95°C for 5 min before being run on a 15% SDS gel to visualize results. Types of IgG used in this assay are described in Table 3.

**TABLE 3:** The manufacturer or purified IgG used in this study

IgG species	Source
Canine IgG	Rockland
Feine IgG	Jackson Immuno Research
Human IgG pooled	Jackson Immuno Research
Human IgG1	SIGMA-ALDRICH
Human IgG2	SIGMA-ALDRICH
Human IgG3	SIGMA-ALDRICH
Human IgG4	SIGMA-ALDRICH
Equine Serum	Horse Clinic, Dept. Vet. Med., FU Berlin
Bovine IgG	SIGMA-ALDRICH
Murine IgG	Sigma Immuno Chemicals
Rabbit IgG	SIGMA-ALDRICH
Goat IgG	SIGMA-ALDRICH
Chicken Serum	Institute of Poultry Diseases, Dept. Vet. Med., FU Berlin

For the cleavage assay performed with protease inhibitors, 1.5 µg IdeC was incubated with different protease inhibitors (2.5 µM) for 30 min at room temperature before the IgG cleavage assay was carried out with 5 µg IgG for 2.5 h at 37°C.

IgG cleavage assays were also performed with variable IdeC concentrations (0.05, 0.1, 0.5, 0.75, 1, 1.5, and 1.75 µg IdeC), with variable incubation times (1, 3, 7, 10, 15, 30, and 60 min). GelQuant.Net 1.8.2 was used to quantify the intensities of the cleavage fragment, and GraphPad Prism 10 was used to plot and statistically analyze these values.

To remove glycoproteins from IgG prior to IgG cleavage assay, New England Biolabs PNGase F was used. Under denaturing conditions, 4 µg purified IgG was added to 1 µL glycoprotein denaturing buffer (10 ×) in 10 µL dH_2_O. The reaction was heated at 100°C for 10 min and chilled on ice for 10 s; 2 µL Glycobuffer 2 (10×), 2 µL 10% NP-40, and 6 µL dH_2_O wewasdded; 1 µL PNGase F was added and mixed gently. The reaction was incubated at 37°C for 1 h. Under non-denaturing conditions, 8 µL IgG was added to 2 µL glycoprotein denaturing buffer (10 ×) in 20 µL dH_2_O; 2 µL PNGase F was added and mixed gently. The reaction was incubated at 37°C for 4–24 h.

### Edman sequencing

Edman sequencing was performed on the cleavage product that resulted from the interaction of IgG and IdeC. The cleavage site was identified by sequence comparison to the amino acid sequence of IgG from the corresponding sequence. Sequencing was performed with cleavage fragments from IgG cleavage assays performed with feline and canine IgG. The IgG assay was prepared as described above and run on a 15% SDS gel to separate the bands. Proteins were transferred to a PVDF membrane by western blot, and the blotted membrane was stained with Ponceau S. The bands of the cleavage products of canine, feline, and human IgG, which were approximately 28 kDa in size, were cut out and dried. Edman sequencing was performed at the Helmholtz Centre for Infection Research, Braunschweig. Geneious 11.1.5 was used to compare the sequences of canine and feline IgG with the sequence produced by the Edman sequencing to identify the cleavage site. Furthermore, a comparison to IgG from other species tested during the host specificity assays was performed to assess whether the cleavage site was conserved.

### Enzyme-linked immunosorbent assay (ELISA)

To determine the binding efficacy of IdeC to feline, canine, and human IgG, an ELISA assay was performed. Plates were coated with increasing amounts of IdeC_C94S (75, 150, 300, 600, 1,000, and 2,000 ng), or positive or negative control, with 0.1 NaHCO_3_ at pH 9.6. The SCM wild-type protein was used as a positive control, at 2,000 ng/well, SCM_Δ173-225_ (A truncated form of the SCM protein, missing the binding center) (49) was used as a negative control at the same concentration. Plates were blocked for 2 h at room temperature with 2.5% BSA in PBST, followed by three washes with 100 µL PBST. The binding capacity of IgG was tested by incubation with HRP-conjugated feline, canine, and human IgG for 2 h. IgG was added at a ratio of 1:2,500. Signal determination was carried out at room temperature with 50 µL tetramethyl bencidinebenzidinee solution (Thermo Fisher Scientific), and the reaction was stopped after 15 min by adding 50 µL 2N H_2_SO_4_. The color reaction was measured using the BioTek SynergyHT Microplate Reader at OD_450nm_.

### Surface plasmon resonance (SPR)

Protein-protein interactions between IdeC and human, feline, and canine IgG were analyzed by SPR using a BIAcore T200 optical biosensor (Cytiva). IgG was immobilized as ligands on a carboxymethyl dextran (CM5) sensor chip using standard amine-coupling procedures as described previously (50). Briefly, IgG was adjusted to 20 µg/mL in 10 mM acetate buffer (pH 4.5 for human and feline IgG, pH 5 for canine IgG) and injected for surface immobilization at a flow rate of 10 µL/min, followed by deactivation of residual activated groups with 1 M ethanolamine. The resonance values of the bound proteins reached ∼2,500 resonance units (RU). The control flow cell was prepared identically but without protein injection. Binding analysis was performed with IdeC_C94Sas analyte (0.3125-10 µg/mL) in PBS containing 0.05% Tween R-20 (PBST) at 25°C using a flow rate of 10 µL/min. Regeneration of the affinity surface was carried out with NaOH. The given RU in the sensograms represents the RU values after subtraction of the values measured in the blank chamber. Each interaction was measured at least three times. Data were analyzed using BIAcore T200 Evaluation Software (version 2.0.1.1). Experimental data were fitted globally using the simple one-step bimolecular association reaction (1:1 Langmuir kinetic: A + B↔AB).

### Crystallization of IdeC

The inactive version of the full-length IdeC protein (30–339 residues, with a substitution of the catalytic Cys94 to Ser) was purified by Ni-NTA affinity chromatography using a 6×His tag. The protein was then concentrated to 9.7 mg/mL with an Amicon Ultra Centrifugal filter device. High-throughput crystallization experiments were conducted using an Oryx-4 robot, and optimal crystals were obtained in a solution of 30% PEG 3350, 200 mM MgCl2, and 100 mM Tris HCl pH 8.5, in a protein:precipitant 1:2 ratio. The truncated version of IdeC (48-339 residues, C94S) was purified using a 6×His tag, followed by a PreScission protease cleavage site. The sample was kept in a buffer of 50 mM Tris HCl pH 7.5 and 100 mM NaCl. For structural determination of the IdeC_T48_C94S structure, co-crystallization experiments were performed with the crystallizable region of IgG (IgG-Fc). The truncated IdeC sample was concentrated to 10 mg/mL and mixed with IgG-Fc in a 1:1 ratio at 4°C for 2 h. The mixture was further concentrated using a 30K cutoff Amicon Ultra Centrifugal filter device before crystallization trials. Crystals were obtained in a solution of 20% PEG 3350, 200 mM Na/K phosphate, and 100 mM Bis Tris propane pH 6.5.

### X-ray data collection, phasing, and model refinement

For both constructs, the crystals were cryo-protected in the crystallization condition supplemented with 20%–30% glycerol. Diffraction data were collected at the ALBA synchrotron XALOC beamline, using a Pilatus 6M detector. Data reduction was performed using XDS (51) and Aimless from the CCP4 suite (52). The molecular-replacement method was employed by using the IdeS structure (PDB 1Y08) as a template. Model refinement and manual model building were performed with Phenix (53), Refmac5 (54), and Coot (55). Data collection and refinement statistics are shown in Table 1.

### Modeling of IdeC-IgG complexes

AlphaFold 3 (26) was employed to model the *S. canis* IdeC complex with full-length canine IgGA (*Canis lupus familiaris*). The IgGA heavy chain sequence used was obtained from GenBank: AAL35301. The VH and CH1 regions were used as a reference for modeling the VL and CL of the light chain. The complex was modeled with a 2:1 ratio of IdeC to IgG, reflecting the presence of two cleavage sites per IgG molecule. Modeling of various IdeC substrates (*Felis catus* IgG1a BAA32229, *Canis lupus familiaris* IgGA AAL35301, and *Homo sapiens* IgG1 P01857) was based on the crystallographic structure of the *S. pyogenes* IdeS:IgG-Fc complex, PDB: 8A47 (25).

## Data Availability

The crystallographic coordinates are deposited in the Protein Data Bank (PDB codes 9HB1 and 9HB2).

## SUPPLEMENTAL MATERIAL

Supplemental figures iai.00248-25-s0001.docx Fig. S1 to S6.

ASM does not own the copyrights to Supplemental Material that may be linked to, or accessed through, an article. The authors have granted ASM a non-exclusive, world-wide license to publish the Supplemental Material files. Please contact the corresponding author directly for reuse.
