# Supplementary material for: Structural and functional characterization of IdeC, a novel IgG-specific protease of Streptococcus canis
Source: Infect Immun. 2025 Jul 31;93(9):e00248-25. doi: 10.1128/iai.00248-25 (PMC12418751; doi:10.1128/iai.00248-25)
Supplement: Supplemental figures — Fig. S1 to S6. [file iai.00248-25-s0001.docx]

**Supplementary figure labels and legends**





Fig S1: **Deglycosylation of canine IgG has no impact on cleavage**.

2 µg of IgG and/or IdeC were used. Reactions were incubated for 3 hours at 37°C and run on a 15% SDS-PAGE gel.


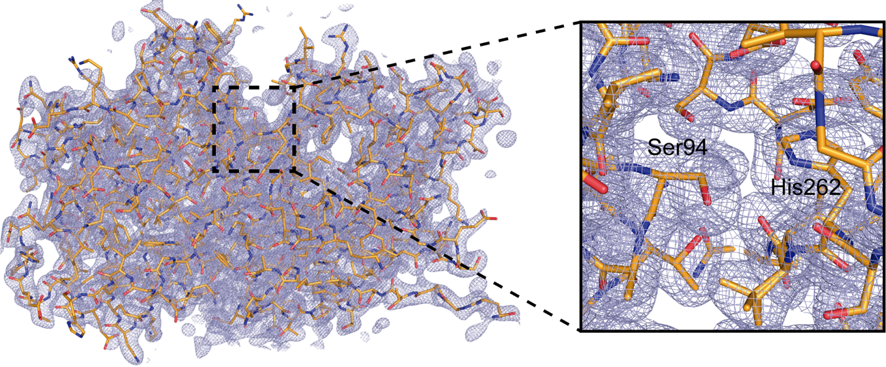


Fig S2**: Quality of the electron density map for the IdeC_C94S_T48 structure.**

The 2Fo-Fc electron density map (contoured at 1 s) is represented as a blue mesh and the protein is shown in yellow stick representation. A close-up view of the active site is highlighted in the inset.


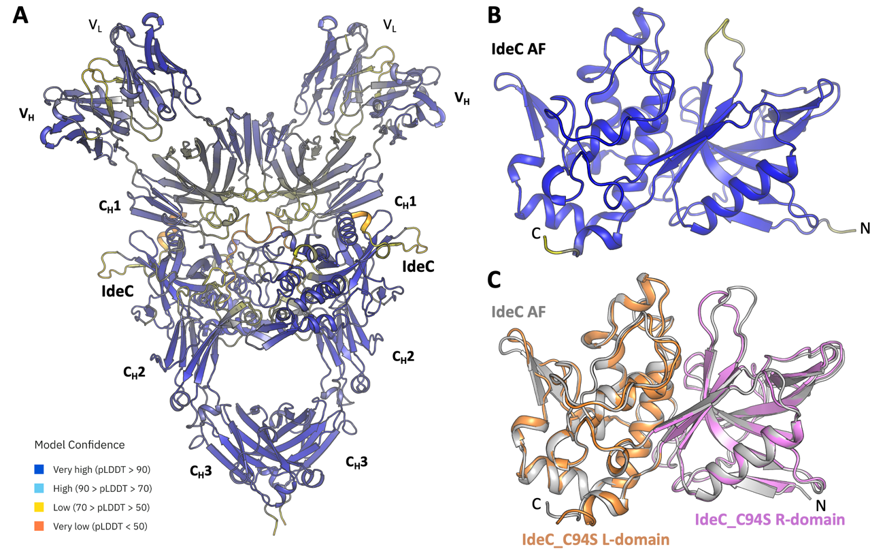


Fig S3: **Computational modeling of the IdeC and IdeC-IgG complex.**

(A) Predicted complex of IdeC with full-length IgGA canine, with high and very high pLDDT (predicted local distance difference test) scores indicating confidence in the model. The complex is shown in cartoon and in same orientation as for Figure 11B. (B) The AlphaFold model for *S. canis* IdeC protein is represented in cartoon, color-coded based on pLDDT scores. (C) A structural comparison between the crystallographic structure of *S. canis* IdeC (displayed in cartoon in the same color scheme as Fig1) and the AlphaFold predicted model (in gray cartoon) is presented.


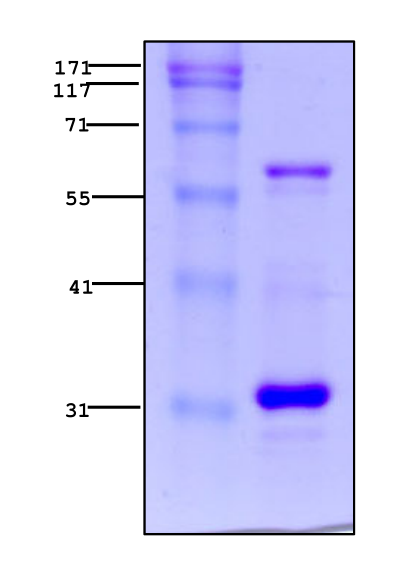


Fig S4: **Non-denatured IdeC.**

Recombinant IdeC was run on an 10 % SDS page gel, without the protein being first denatured by incubation at 95 °C for 10 min. This assay was also carried out under non-reducing conditions using Laemmli buffer without DTT, the reducing agent. Here we see two bands instead of one; the usual ~35 kDa and seemingly a dimer at ~60 kDa.





Fig S5: **IdeC cleaves different human IgG sub types with varying efficiency.**

The most effective cleavage occurs in IgG3, followed by IgG1, IgG4 and lastly IgG2. 2 µg of IgG and IdeC each were used in the assay. Reactions were incubated for 3 hours at 37°C and then run on a 15% SDS-PAGE gel.


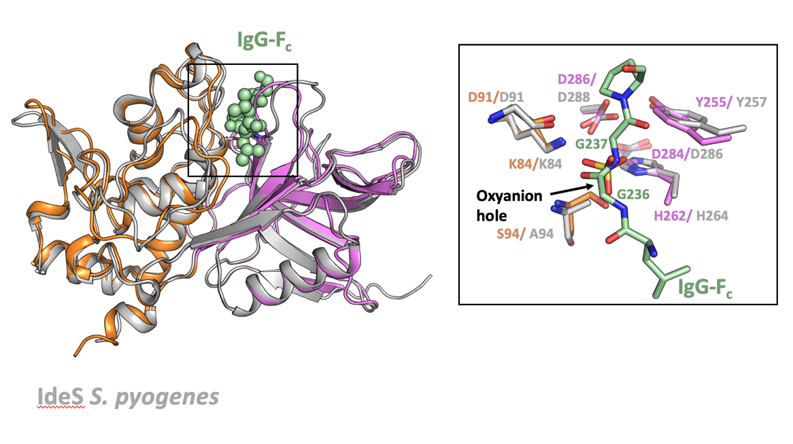


Fig S6**: Structural comparison of *S. canis* IdeC (cartoon with the same colour scheme as in Fig 10) with *S. pyogenes* IdeS (grey cartoon) (PDB: 8A47).**

The IgG-Fc fragment is depicted as green spheres. In a frame, a detailed view shows the superimposed active sites of the proteins, with the IgG-Fc fragment illustrated as green sticks.
